# Optimizing oral antibiotic prescribing at hospital discharge: a single center, quasi-experiment pilot study

**DOI:** 10.1017/ash.2025.10061

**Published:** 2025-06-30

**Authors:** Ahmad Aloufi, Calvin Ka-Fung Lo, Colin Lee, Adrianna Gunton, Victor Leung

**Affiliations:** 1 Antimicrobial Stewardship Program, Providence Health Care, Vancouver, BC, Canada; 2 Department of Pathology and Laboratory Medicine, University of British Columbia, Vancouver, BC, Canada; 3 Division of Infectious Diseases, University of British Columbia, Vancouver, BC, Canada; 4 Division of Medical Microbiology and Virology, St Paul’s Hospital, Providence Health Care, Vancouver, BC, Canada; 5 Division of Infectious Diseases, Department of Medicine, Providence Health Care, Vancouver, BC, Canada

## Abstract

Our quasi-experimental pilot study between July to September 2024 showed that real-time audit/feedback for antibiotic discharge prescriptions improved appropriateness from 50% to 83%, while decreasing median antibiotic duration compared to preintervention period. Hospital discharges are an important transition point for antimicrobial stewardship interventions at discharge.

## Introduction

Transition to oral (PO) antibiotics helps facilitate hospital discharges for common infections including urinary tract infections (UTI), skin and soft tissue infections (SSTI) and pneumonia. However, prescriptions at discharge can contribute to excess total therapy durations.^1^ Audit and feedback is an effective intervention, but many hospitals do not have integrated work flows to routinely identify patients being discharged on PO antibiotics.^2–10^

Our 3-month pilot study was conducted at a tertiary care teaching hospital in Vancouver, British Columbia, to evaluate whether active audit and feedback on discharge prescriptions improves antibiotic prescription practices for patients discharged from Internal Medicine Clinical Teaching Unit (CTU).

## Methods

Our retrospective cohort consisted of CTU patient discharges from a point prevalence survey between October 1st, 2023 to March 31st, 2024 (preintervention). Our intervention involved prospective real-time audit and feedback for patients discharged and prescribed PO antibiotics between July 15, 2024 to September 20, 2024.

Our AMS team (AA, CL and VL) was informed of upcoming discharges by a member of each CTU team, via a CTU discharge Signal® group chat. All CTU patients discharged on PO antibiotics were included. We excluded patients discharged on prophylactic antibiotics, suppressive antibiotics, topical antibiotics, discharges to Outpatient Parenteral Antimicrobial Therapy, transfers to other acute medical facilities, and patient-initiated discharges. Based on concordance with local treatment guidelines, documented AMS intervention notes were detailed in electronic health record if either duration or choice of antibiotic was considered inappropriate. Discharges not reviewed by AMS team (ie, weekend, holiday, or overnight discharges, missed notification) were included as a subgroup of prospective cases without AMS intervention.

Pertinent variables included infectious syndrome treated and intended antibiotic prescription (including dose, effective therapy duration and intended duration upon discharge). Each antibiotic could have up to two interventions (duration and choice). For patients discharged on multiple antibiotics, interventions were counted for each antibiotic. Lastly, patients evaluated by specialist services (ie, Infectious Diseases (ID) or Respirology) were considered as “appropriate” unless the patient was discharged with antibiotics different from their treatment recommendations.

The primary outcome was appropriateness of antibiotic choice and duration based on local hospital guidelines. In cases without applicable local guidelines, appropriateness was then based on AMS physician review. Subgroup analyses were conducted only in patients with uncomplicated infections including community-acquired pneumonia (CAP), UTI (including pyelonephritis) and SSTIs.

Secondary outcomes included (i) median duration of discharge therapy (counting from discharge date, in days); (ii) total therapy duration (from start date, in days); (iii) acceptance rate for intervention.

Metrics between both periods were compared with the Pearson *χ* ^2^ or Wilcoxon rank-sum test, with *P*-values < .05 being considered statistically significant (SPSS version 29.0.2).

## Results

Between October 1, 2023–March 31, 2024 (preintervention), 158 of 548 patients were discharged from CTU on PO antibiotics (total of 181 prescriptions). During the intervention period (July 15–September 20, 2024), 121 patients were discharged on oral antibiotics (total of 133 prescriptions). Of these 121 patients, 44 patients were discharged without AMS review (Supplementary Figure 1).

Postintervention, only 58 patients were considered amenable for intervention as further 19 patients had specialist consultation (ie, 14 by Infectious Diseases, and 5 by Respirology). The three most frequently used PO antibiotics were amoxicillin-clavulanate (35/122, 29%), cefuroxime (15/122, 12%), and cephalexin (15/122, 12%) (Supplementary Table 1). The most common infectious syndromes were SSTI (22%), CAP (17%) and UTI (16%) (Supplementary Table 2).

Appropriateness of discharge prescriptions increased from 50% preintervention to 83% postintervention (*P* < .001). Acceptance rate was high overall at 85%, which included acceptance of the intervention in 26/31 cases for duration, and 9/10 cases for antibiotic choice.

The median number of prescription days from discharge was 5 days [IQR 3–10] in the preintervention versus 4 days [IQR 2–6] for the postintervention period (*P* < .001). Meanwhile, median total antibiotic duration was 9 days [IQR 7–14] preintervention versus 7 days [IQR 5–10] postintervention (*P* = .012) (Table 1).

**Table 1.** Median treatment duration (days) for pre and postintervention, including full analysis and sub analysis

Patient cohort	Duration	Preintervention	Postintervention	P-value
Full analysis^a^ *n = 181 antibiotics (pre)* *133 antibiotics (post)*	Discharge^b^	5 [IQR 3–10]	4 [IQR 2–6]	**<.001**
	Total^c^	9 [IQR 7–14]	7 [IQR 5–10]	**.012**
Subgroup analysis^d^ *n = 116 antibiotics (pre)* *100 antibiotics (post)*	Discharge	4 [IQR 2–7]	3 [IQR 2–5]	**.003**
	Total	7 [IQR 5–10]	7 [IQR 5–9]	.052
CAP/COPDe/HCAP *n = 69 antibiotics (pre)* *43 antibiotics (post)*	Discharge	3 [IQR 2–5]	2 [IQR 1–4]	**.023**
	Total	6 [IQR 5–7]	5 [IQR 5–7]	**.039**
Cystitis/Pyelonephritis *n = 23 antibiotics (pre)* *23 antibiotics (post)*	Discharge	7 [IQR 4–9]	4 [IQR 3–5]	**.016**
	Total	12 [IQR 7–14]	8 [IQR 7–10]	.054
SSTI *n = 27 antibiotics (pre)* *35 antibiotics (post)*	Discharge	5 [IQR 3–7]	3 [IQR 1–5]	**.01**
	Total	10 [IQR 9–12]	7 [IQR 7–10]	**<.001**

Abbreviations: CAP, community acquired pneumonia; COPDe, Chronic Obstructive Pulmonary Disease Exacerbation; HCAP, Health Care Associated pneumonia; IQR, Interquartile Range; SSTI, Skin and Soft Tissue Infection;

a All patients included in the analysis.

b Duration of prescribed antibiotics on discharge.

c Duration of total therapy patient received as inpatient and on discharge.

d Sub-analysis for patients with uncomplicated CAP, HACP, COPDe, cystitis, pyelonephritis or SSTI with or without bacteremia.

In the subgroups of uncomplicated pneumonia (*n* = 112), UTI (*n* = 46), and SSTI (*n* = 62), there was a statistically significant increase in appropriate prescriptions between preintervention and postintervention period (41% vs 81%, *P* < .01) (Table 2).

**Table 2.** Appropriateness and interventions per infectious syndromes (pre and postintervention)

Syndrome	Preintervention			Postintervention			
	Antibiotics (number)	Appropriate	Inappropriate	Antibiotics (number)	Appropriate	Inappropriate	Interventions (Accepted)
Respiratory ^a^	69	33 (48%)	36 (52%)	43	36 (84%)	7 (16%)	12 (11)
UTI ^b^	23	9 (39%)	14 (61%)	23	18 (78%)	6 (12%)	6 (4)
SSTI	27	7 (26%)	20 (74%)	35	29 (83%)	6 (17%)	19 (17)

Abbreviations—SSTI, Skin and soft tissue infection; UTI, urinary tract infection;

a Includes community acquired pneumonia, healthcare associated pneumonia and chronic obstructive pulmonary disease exacerbation.

b Includes cystitis, pyelonephritis and asymptomatic bacteriuria.

## Discussion

Similar to previous studies,^6,8^ approximately 50% of antibiotic prescriptions at discharge were deemed to be inappropriate prior to our intervention. Oral antibiotics are typically not targets for AMS intervention and so they are often prescribed by CTU providers without AMS team review and input. Even though CTU teams typically have clinical pharmacists available to review discharge prescriptions, high patient turnover and workload may result in lower prioritization of antimicrobial stewardship interventions. Twelve (36%) out of 31 interventions for duration were completed on patients who did not require further antibiotics. We observed that CTU physicians followed fixed longer durations (eg, 5 d for CAP instead of 3, and 10 d for SSTI instead of 5) without considering clinical improvement. Another recurrent reason for prolonged antibiotics at discharge was using preset order durations (eg, 5 d for pneumonia regardless of previous duration) as previously reported.^7^ Despite available guidelines for uncomplicated infectious syndromes, inappropriateness was still observed in the preintervention phase regardless of infection type (Table 2).

This study showed that timely antimicrobial stewardship interventions at discharge led to a decrease in total antibiotic treatment duration. Median durations for respiratory (including healthcare-associated pneumonia) and SSTI were significantly decreased for both discharge and total therapy, whereas with UTI, no statistical difference was observed in total therapy duration between both periods (Table 1).

This study has several limitations. The method for identifying planned discharges did not factor for delayed discharges (where patient completes antibiotic course in hospital) or unexpected premature discharges (which were not communicated via Signal group chat). Secondly, ID and Respirology consultations were automatically considered as appropriate prescriptions since these cases had been historically excluded from AMS review. Thirdly, assessment of appropriateness could be investigator-dependent for cases where guidelines are not applicable. Fourthly, both periods had different durations (ie, 6-month preintervention followed by our 3-month pilot intervention) due to limited staff and lack of funding to sustain this intervention to equal length. Finally, there were differences in syndrome distribution between periods which may have affected duration and appropriateness (eg, the greater prevalence of respiratory infections preintervention compared to our intervention period).

Our study demonstrates the effectiveness of a timely and structured AMS intervention in improving antibiotic appropriateness at discharge. These findings emphasize a need for effective and systematic approaches to ensure antibiotics are prescribed appropriately on discharge.

## Supporting information

10.1017/ash.2025.10061.sm001 Aloufi et al. supplementary material 1 Aloufi et al. supplementary material

10.1017/ash.2025.10061.sm002 Aloufi et al. supplementary material 2 Aloufi et al. supplementary material

10.1017/ash.2025.10061.sm003 Aloufi et al. supplementary material 3 Aloufi et al. supplementary material

## Supplementary material

The supplementary material for this article can be found at https://doi.org/10.1017/ash.2025.10061
