# Supplementary material for: Optimizing oral antibiotic prescribing at hospital discharge: a single center, quasi-experiment pilot study
Source: Antimicrob Steward Healthc Epidemiol. 2025 Jun 30;5(1):e147. doi: 10.1017/ash.2025.10061 (PMC12224137; doi:10.1017/ash.2025.10061)
Supplement: Aloufi et al. supplementary material 1 — Aloufi et al. supplementary material [file S2732494X25100612sup001.pdf]

Supplementary Figure 1: Flow chart of patients reviewed and eligible for analysis, pre- and post-intervention

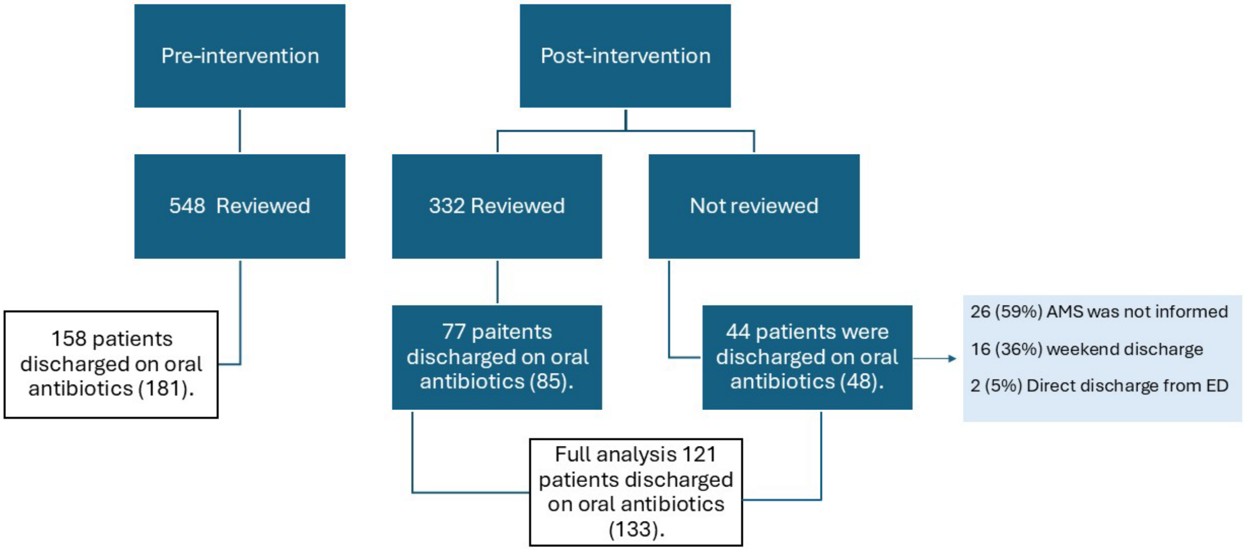

Figure caption: Flow chart with breakdown of pre- and post-intervention cohorts. Numbers outside of parentheses are patient counts per group. Parentheses indicate number of antimicrobial agents (some patients were prescribed >1 antibiotic). *White boxes*: number of patients with oral antibiotics listed on their discharge prescription and included for our analysis (i.e., 158 pre-intervention, 121 post-intervention). *Light blue box, right*: breakdown of patients not reviewed by the antimicrobial stewardship team, but included as a subgroup in our analysis (**Methods** section for full details). *Abbreviations*: AMS = antimicrobial stewardship; ED = Emergency Department.
