# Supplementary material for: Optimizing oral antibiotic prescribing at hospital discharge: a single center, quasi-experiment pilot study
Source: Antimicrob Steward Healthc Epidemiol. 2025 Jun 30;5(1):e147. doi: 10.1017/ash.2025.10061 (PMC12224137; doi:10.1017/ash.2025.10061)
Supplement: Aloufi et al. supplementary material 2 — Aloufi et al. supplementary material [file S2732494X25100612sup002.pdf]

Supplementary Table 1: Distribution of oral antimicrobials prescribed at discharge (n = 122)

Antibiotics used at discharge	Total Count (%)[a]
Amoxicillin-clavulanate	35 (29%)
Cephalexin	15 (12%)
Cefuroxime	15 (12%)
Ciprofloxacin	13 (11%)
Amoxicillin	8 (7%)
Cefadroxil	7 (6%)
Doxycycline	5 (4%)
Trimethoprim / Sulfamethoxazole	5 (4%)
Moxifloxacin	4 (3%)
Others [b]	15 (12%)

[a] Patients can also be discharged on >1 antimicrobial agent

[b] Others: oral vancomycin, azithromycin, levofloxacin, cefixime, tetracycline and metronidazole (part of *H. pylori* regimen), fosfomycin.
