# Supplementary material for: Optimizing oral antibiotic prescribing at hospital discharge: a single center, quasi-experiment pilot study
Source: Antimicrob Steward Healthc Epidemiol. 2025 Jun 30;5(1):e147. doi: 10.1017/ash.2025.10061 (PMC12224137; doi:10.1017/ash.2025.10061)
Supplement: Aloufi et al. supplementary material 3 — Aloufi et al. supplementary material [file S2732494X25100612sup003.pdf]

Table 2: Distribution of infectious syndromes amidst patient cohort

Syndrome	No. (%)[a]
SSTI	31 (22%)
CAP	24 (17%)
Bacteremia	18 (13%)
UTI<br>- Cystitis<br>- Pyelonephritis<br>- Asymptomatic bacteriuria	23 (16%)<br>- 14 (10%)<br>- 8 (6%)<br>- 1 (0.7%)
COPD exacerbation	7 (5%)
HCAP	5 (4%)
Empyema/complicated pneumonia	4 (3%)
Intra-abdominal infection<br>- Intra-abdominal abscess<br>- Cholangitis/Cholecystitis	13 (9%)<br>- 7 (3%)<br>- 6 (4%)
OM/ Septic Arthritis	4 (3%)
Clostridioides difficile infection	3 (2%)
Others [b]	8 (6%)

Abbreviations - CAP: community acquired pneumonia, COPD: chronic obstructive pulmonary disease, HCAP: health care associated pneumonia, OM: osteomyelitis. SSTI: Skin and soft tissue infection, UTI: urinary tract infection.

[a] Some patients had > 1 concurrent infectious syndrome (e.g., pneumonia with cellulitis).

[b] Others: gastroenteritis, otitis media, *H. pylori*, sepsis without clear focus.
